# Predicted effects of the introduction of long-acting injectable cabotegravir pre-exposure prophylaxis in sub-Saharan Africa: a modelling study

**DOI:** 10.1016/S2352-3018(22)00365-4

**Published:** 2023-01-12

**Authors:** Jennifer Smith, Loveleen Bansi-Matharu, Valentina Cambiano, Dobromir Dimitrov, Anna Bershteyn, David van de Vijver, Katharine Kripke, Paul Revill, Marie-Claude Boily, Gesine Meyer-Rath, Isaac Taramusi, Jens D Lundgren, Joep J van Oosterhout, Daniel Kuritzkes, Robin Schaefer, Mark J Siedner, Jonathan Schapiro, Sinead Delany-Moretlwe, Raphael J Landovitz, Charles Flexner, Michael Jordan, Francois Venter, Mopo Radebe, David Ripin, Sarah Jenkins, Danielle Resar, Carolyn Amole, Maryam Shahmanesh, Ravindra K Gupta, Elliot Raizes, Cheryl Johnson, Seth Inzaule, Robert Shafer, Mitchell Warren, Sarah Stansfield, Roger Paredes, Andrew N Phillips

**Affiliations:** Institute for Global Health, University College London, London, UK (J Smith PhD, L Bansi-Matharu PhD, V Cambiano PhD, Prof M Shahmanesh PhD, Prof A N Phillips PhD); Partners in Hope, Lilongwe, Malawi (J J van Oosterhout PhD); Department of Medicine, David Geffen School of Medicine (J J van Oosterhout, Prof R J Landovitz MD) and Center for Clinical AIDS Research and Education (Prof R J Landovitz), University of California, Los Angeles, CA, USA; Division of Infectious Diseases, Brigham and Women’s Hospital, Boston, MA, USA (Prof D Kuritzkes MD); Department of Medicine, Harvard Medical School, Boston, MA, USA (Prof D Kuritzkes, M J Siedner MD); Global HIV, Hepatitis, and STIs Programmes, WHO, Geneva, Switzerland (R Schaefer PhD, C Johnson PhD, S Inzaule PhD); Division of Infectious Diseases, Massachusetts General Hospital, Boston, MA, USA(M J Siedner); Clinical Research Department, Africa Health Research Institute, Mtubatuba, South Africa (Prof M Shahmanesh, Prof R K Gupta PhD, M J Siedner); National Hemophilia Center, Sheba Medical Center, Ramat Gan, Israel (J Schapiro MD); Wits RHI (Prof S Delaney-Moretlwe PhD) and Health Economics and Epidemiology Research Office (HE2RO), Department of Internal Medicine, School of Clinical Medicine (G Meyer-Rath PhD) and Ezintsha (Prof F Venter PhD), Faculty of Health Sciences, University of the Witwatersrand, Johannesburg, South Africa; Johns Hopkins University School of Medicine and Bloomberg School of Public Health, Johns Hopkins University, Baltimore, MD, USA (Prof C Flexner MD); Tufts Medical Center, Tufts University School of Medicine, Boston, MA, USA (M Jordan MD); Department of Viroscience, Erasmus Medical Centre, Rotterdam, Netherlands (D van de Vijver PhD); Avenir Health, Takoma Park, MD, USA (K Kripke PhD); Vaccine and Infectious Disease Division (S Stansfield PhD) and Public Health Sciences Division, Fred Hutchinson Cancer Center, Seattle, WA, USA (D Dimitrov PhD); Centre for Health Economics, University of York, York, UK (Prof P Revill MSc); MRC Centre for Global Infectious Disease Analysis, School of Public Health, Imperial College London, London, UK (Prof M-C Boily PhD); Department of Population Health, New York University Grossman School of Medicine, New York, NY, USA (A Bershteyn PhD); National AIDS Council, Harare, Zimbabwe (I Taramusi MPH); Department of Clinical Medicine, Rigshospitalet, University of Copenhagen, Copenhagen, Denmark (Prof J D Lundgren DSc); Regional Office for Africa, WHO, Gauteng, South Africa (M Radebe PhD); Infectious Diseases Program, Clinton Health Access Initiative, New York, NY, USA (D Ripin PhD, S Jenkins BSc, D Resar MSc, C Amole BA); Department of Medicine, University of Cambridge, Cambridge, UK (Prof R K Gupta); US Department of Health and Human Services, Centers for Disease Control, Atlanta, GA, USA (E Raizes MD); Department of Medicine, Stanford University, Palo Alto, CA, USA (R Shafer MD); AVAC, New York, NY, USA (M Warren BA); Department of Infectious Diseases, Irsi Caixa Institut de Recerca de la SIDA, Barcelona, Spain (R Paredes PhD)

## Abstract

**Background:**

Long-acting injectable cabotegravir pre-exposure prophylaxis (PrEP) is recommended by WHO as an additional option for HIV prevention in sub-Saharan Africa, but there is concern that its introduction could lead to an increase in integrase-inhibitor resistance undermining treatment programmes that rely on dolutegravir. We aimed to project the health benefits and risks of cabotegravir-PrEP introduction in settings in sub-Saharan Africa.

**Methods:**

With HIV Synthesis, an individual-based HIV model, we simulated 1000 setting-scenarios reflecting both variability and uncertainty about HIV epidemics in sub-Saharan Africa and compared outcomes for each with and without cabotegravir-PrEP introduction. PrEP use is assumed to be risk-informed and to be used only in 3-month periods (the time step for the model) when having condomless sex. We consider three groups at risk of integrase-inhibitor resistance emergence: people who start cabotegravir-PrEP after (unknowingly) being infected with HIV, those who seroconvert while on PrEP, and those with HIV who have residual cabotegravir drugs concentrations during the early tail period after recently stopping PrEP. We projected the outcomes of policies of cabotegravir-PrEP introduction and of no introduction in 2022 across 50 years. In 50% of setting-scenarios we considered that more sensitive nucleic-acid-based HIV diagnostic testing (NAT), rather than regular antibody-based HIV rapid testing, might be used to reduce resistance risk. For cost-effectiveness analysis we assumed in our base case a cost of cabotegravir-PrEP drug to be similar to oral PrEP, resulting in a total annual cost of USD$144 per year ($114 per year and $264 per year considered in sensitivity analyses), a cost-effectiveness threshold of $500 per disability-adjusted life years averted, and a discount rate of 3% per year.

**Findings:**

Reflecting our assumptions on the appeal of cabotegravir-PrEP, its introduction is predicted to lead to a substantial increase in PrEP use with approximately 2·6% of the adult population (and 46% of those with a current indication for PrEP) receiving PrEP compared with 1·5% (28%) without cabotegravir-PrEP introduction across 20 years. As a result, HIV incidence is expected to be lower by 29% (90% range across setting-scenarios 6–52%) across the same period compared with no introduction of cabotegravir-PrEP. In people initiating antiretroviral therapy, the proportion with integrase-inhibitor resistance after 20 years is projected to be 1·7% (0–6·4%) without cabotegravir-PrEP introduction but 13·1% (4·1–30·9%) with. Cabotegravir-PrEP introduction is predicted to lower the proportion of all people on antiretroviral therapy with viral loads less than 1000 copies per mL by 0·9% (–2·5% to 0·3%) at 20 years. For an adult population of 10 million an overall decrease in number of AIDS deaths of about 4540 per year (−13 000 to −300) across 50 years is predicted, with little discernible benefit with NAT when compared with standard antibody-based rapid testing. AIDS deaths are predicted to be averted with cabotegravir-PrEP introduction in 99% of setting-scenarios. Across the 50-year time horizon, overall HIV programme costs are predicted to be similar regardless of whether cabotegravir-PrEP is introduced (total mean discounted annual HIV programme costs per year across 50 years is $151·3 million *vs* $150·7 million), assuming the use of standard antibody testing. With antibody-based rapid HIV testing, the introduction of cabotegravir-PrEP is predicted to be cost-effective under an assumed threshold of $500 per disability-adjusted life year averted in 82% of setting-scenarios at the cost of $144 per year, in 52% at $264, and in 87% at $114.

**Interpretation:**

Despite leading to increases in integrase-inhibitor drug resistance, cabotegravir-PrEP introduction is likely to reduce AIDS deaths in addition to HIV incidence. Long-acting cabotegravir-PrEP is predicted to be cost-effective if delivered at similar cost to oral PrEP with antibody-based rapid HIV testing.

**Funding:**

Bill & Melinda Gates Foundation, National Institute of Allergy and Infectious Diseases of the National Institutes of Health.

## Introduction

HIV incidence remains high in many parts of sub-Saharan Africa, with around 900 000 new infections in 2020.^1^ Pre-exposure prophylaxis (PrEP) has the potential to substantially reduce HIV incidence but studies done in the region on use of oral PrEP have shown low continuation and adherence.^2,3^ A preference for long-acting HIV prevention products has been reported,^4,5^ suggesting that availability of such products could increase the uptake of PrEP. In two trials, the HIV Prevention Trials Network 083^6^ and 084,^7^ long-acting cabotegravir PrEP injections every 2 months were safe and substantially lowered HIV incidence. However, cabotegravir is an integrase-inhibitor that is similar to dolutegravir, part of the first-line HIV treatment recommended by WHO. There is concern about development of resistance to integrase-inhibitor drug resistance when people with HIV are exposed to cabotegravir-PrEP, which could confer cross-resistance to dolutegravir and undermine the effects of treatment. Individuals might have HIV while having cabotegravir present in three main contexts: initiating PrEP when recent HIV infection is present but not yet detected because HIV test sensitivity is below 100%;^8^ acquiring HIV while on PrEP because prevention efficacy is likely below 100%;^6^ or acquiring HIV after having stopped PrEP injections but while residual cabotegravir has not completely been eliminated from the system (ie, the tail period of elimination).^6,7,9^ Indeed, resistance has occurred in cabotegravir-PrEP trials.^10–13^ The question thus arises whether the benefits of cabotegravir-PrEP introduction on HIV incidence outweigh any negative effects due to the development of resistance to dolutegravir. Nucleic-acid-based HIV diagnostic testing (hereafter referred to as NAT) has been proposed in addition to rapid antibody-based testing (hereafter referred to as antibody testing) as a more sensitive alternative to antibody testing alone to minimise risks of drug resistance in the first two contexts, but this approach would have substantial implementation and cost implications. We used an existing individual-based model to quantify these trade-offs in the context of settings in sub-Saharan Africa, and to assess the cost-effectiveness of cabotegravir-PrEP introduction.

## Methods

### Model description and analysis approach

Methods are detailed in the appendix, and here we provide a summary. HIV Synthesis is an individual-based simulation model, which has been described previously.^14–16^ Each model run generates a simulated population of adults from 1989 (taken as the start of the epidemic) with variables updated every 3 months, including age, sex, primary and non-primary condomless sex partners, whether currently a female sex worker, HIV testing, male circumcision status, presence of sexually transmitted infections other than HIV, and use of oral PrEP. Only heterosexual sex, the main driver of the epidemic, is modelled. In people positive for HIV, we model viral load, CD4 cell count, use of specific antiretroviral drugs, and drug resistance. Risk of AIDS death in the model depends on the current CD4 cell count, viral load, age, and antiretroviral therapy (ART) status. For a person on treatment, viral load, CD4 cell count, and risk of resistance are primarily determined by the adherence, drug concentration, and the number of active drugs being taken, of which the activity rate of each drug depends on its underlying potency and which, if any, drug-resistance mutations are present (appendix p 39).

Through sampling of parameter values (appendix pp 71–87) at the start of each model run we created 1000 setting-scenarios reflecting uncertainty in assumptions and a range of characteristics similar to those seen in sub-Saharan Africa (table 1). For each setting-scenario, we consider the situation in the third quarter of 2022 (the start date we decided on a priori) and make a pairwise comparison of outcomes of two policies: introduction and scale-up over 2 years of cabotegravir-PrEP (without restriction by age or gender) then continuation for 50 years, and no introduction of cabotegravir-PrEP. We show results for the effects of the policies over 20 years and 50 years for the purposes of assessing long-term effectiveness and cost-effectiveness. All model outputs are reported as means and 90% ranges across setting-scenarios.

For the integrase-inhibitors, dolutegravir and cabotegravir,^18–26^ we consider mutations at codon positions 118, 140, 148, 155, and 263, assumed to lead to a resistance level of 0·75 (80%)/1·00 (20%) on a scale of 0 to 1. Throughout, multiple parameter values are separated by a /, which means that one of the values is sampled at random for each model run (ie, setting-scenario), and the percentage chance of being selected is given in brackets if not equally likely. The amount of resistance together with the underlying drug potency determines the amount of activity of the drug. If the infecting virus includes such a mutation, it is transmitted with probability 0·2/0·4/0·6/0·8, reflecting high uncertainty over transmissibility.^25,26^

PrEP is modelled as described in the appendix (pp 33–37). We again hypothesise that PrEP will be used in a risk-informed way: only in 3-month periods when there is an indication for PrEP. Indications for PrEP were having had condomless sex with at least one short-term partner or being concerned that they have a long-term partner who has HIV but is not on ART.^14^ We assume an oral PrEP efficacy of 90%/95% per infected condomless partner per 3 months.^3^ The amount of adherence for an individual in a given 3-month period is quantified on a scale of 0–1. Our assumptions result in a mean proportion of people with high (ie, more than 80%) adherence to oral PrEP of 86% (34–92%), and mean effectiveness (ie, efficacy × adherence) of 71%.

Cabotegravir-PrEP efficacy is also assumed to be 90%/95%;^6,7,27^ although we acknowledge that there are no direct efficacy data for protection for men who have sex with women and for a person on cabotegravir-PrEP the drug concentration has a value of 1 so the effectiveness equals the efficacy. We assume in most setting scenarios that cabotegravir-PrEP is more widely appealing than oral PrEP and so its introduction results in an increase in the overall number of people taking PrEP and a decrease in the number of people taking oral PrEP (table 2; appendix p 35). Cabotegravir-PrEP is administered every 2 months, so our 3-month model time step means that we consider discrete periods of cabotegravir use of 3 months at a time.

For each integrase-inhibitor resistance mutation, the risk that it arises in the initial 3–6 month period of infection for a person on cabotegravir-PrEP is 0·1/0·2/0·3/0·5 per mutation. For a person in the cabotegravir-PrEP tail period this risk is 0·00/0·05/0·75/1·00/1·33 times this per-mutation probability, reflecting uncertainty since lower drug concentrations lead to less viral suppression but less selection pressure than higher concentrations. The wide variation in resistance risk across setting-scenarios reflects uncertainty due to limited data on emergence of resistance from clinical trials (in the HPTN 083 and HPTN 084 trials, one person with integrase-inhibitor resistance of five people initiating cabotegravir-PrEP while having HIV and four people with integrase-inhibitor resistance of five people with breakthrough infections)^10–13^ as well as whether there could be some suboptimal cabotegravir-PrEP injecting in routine practice.

For a person who has HIV detected when they are receiving cabotegravir-PrEP or in the early tail, we assume in 20% of setting-scenarios that initial ART will consist of tenofovir disoproxil fumarate–lamivudine and ritonavir-boosted atazanavir rather than dolutegravir. We assume that viral load monitoring tests are done in a period when they are due (or overdue) with probability of 0·0 (5%)/0·1 (30%)/0·7 (50%)/1·0 (15%), and that switching to a ritonavir-boosted-atazanavir-based regimen occurs with probability of 0·10/0·20/0·50/1·00 per 3 months in a person with confirmed virological failure. Cabotegravir-PrEP is assumed to be less efficacious in preventing HIV acquisition (by 0·25/0·50/0·75-fold, sampled with equal probability) when the sexual partner with HIV to whom the subject is exposed carries virus with an integrase-inhibitor resistance mutation.

To allow us to understand the extent to which effects of cabotegravir-PrEP on integrase-inhibitor drug resistance influences the overall effect of its introduction, we ran an additional set of setting-scenarios in which cabotegravir-PrEP was assumed to not lead to integrase-inhibitor resistance.

Sensitivity of HIV tests according to time from infection and exposure to cabotegravir-PrEP are shown in the appendix (p 36), and show that cabotegravir exposure reduces test sensitivity and this sensitivity is partly improved by use of NAT rather than antibody testing. There remains substantial uncertainty, reflected in the range of values sampled.

### Costs, long-term health outcomes, and cost-effectiveness

As before,^14^ in our base case we used a cost of oral PrEP provision of USD$116 per year, consisting of a drug cost of $60 (including supply chain costs, based on the South Africa tender price for PrEP drugs),^28^ $4 per 3 months for an antibody-test, and $10 per 3 months for additional costs necessary to facilitate education and access. These additional costs depend on the delivery approach; in our primary analysis, we used a cost similar to that used in a cost-effectiveness evaluation in South Africa.^28^

For cabotegravir-PrEP we used a cost of $144 per year. The drug cost is assumed to be similar to that of oral PrEP ($60 per year), which is above the estimated production cost.^29^ In sensitivity analyses we consider a halving of this drug cost, giving a total cost of $114, and a doubling of both drug cost and clinic visit costs (ie, the additional costs needed to facilitate education and access) for cabotegravir-PrEP, giving a total of $264. HIV test and clinic visit costs are 1·5 times those of oral PrEP as it requires six visits per year rather than four. ART drug (dolutegravir, tenofovir, and lamivudine) costs are $65 annually (including supply chain). NAT tests are assumed to cost the same as viral load tests (ie, $22).

We simulate the absolute numbers of health-related events, costs, and disability-adjusted life years (DALYs) for a base population of 10 million adults in 2022 over a 50-year period. Resource use and cost were analysed from a health-care system perspective. We also calculate net DALYs, a measure of the full health implications of the intervention being delivered by the health-care system, accounting for opportunity costs.^30^ We use a cost-effectiveness threshold of $500 per DALY averted and a 3% discount rate for both costs and health outcomes to calculate net DALYs averted; if net DALYs being averted means that the incremental cost effectiveness ratio is less than $500. Country-specific thresholds are uncertain but $500 per DALY averted is likely to be at the upper end on the basis of evidence concerning how resources would otherwise be used.^31^ Additional costing information is included in the appendix (p 88).

### Role of the funding source

The funders of the study had no role in the study design, data collection, data analysis, data interpretation, or writing of the report.

## Results

Given the assumed uptake, cabotegravir-PrEP introduction results in a mean reduction of 29% (90% range across setting scenarios 6–52%) in HIV incidence over 20 years (table 2; figure) and a 26% (4–47%) reduction in prevalence. With antibody testing, 0·67% (90% range across setting scenarios 0·07–1·87%) of people on cabotegravir-PrEP at any point in time are predicted to have HIV, compared with (0·16% (0·02–0·51%) with NAT (table 3). In the context of a population of 10 million people older than 15 years, with a mean of 192 000 (90% range across setting scenarios 55 000–480 000) on cabotegravir-PrEP (table 3), the mean number of people with HIV initiating or re-starting cabotegravir-PrEP per year is estimated at 960 (88–3172) with antibody testing and 472 (44–1548) if NAT is used at initiation and reinitiation. Accounting also for breakthrough infections, a mean of 2020 (90% range across setting scenarios 164–6416) people per year are predicted to newly develop integrase-inhibitor resistance while on cabotegravir-PrEP by use of antibody testing compared with 1020 (104–4160) by NAT.

**Figure 1:**
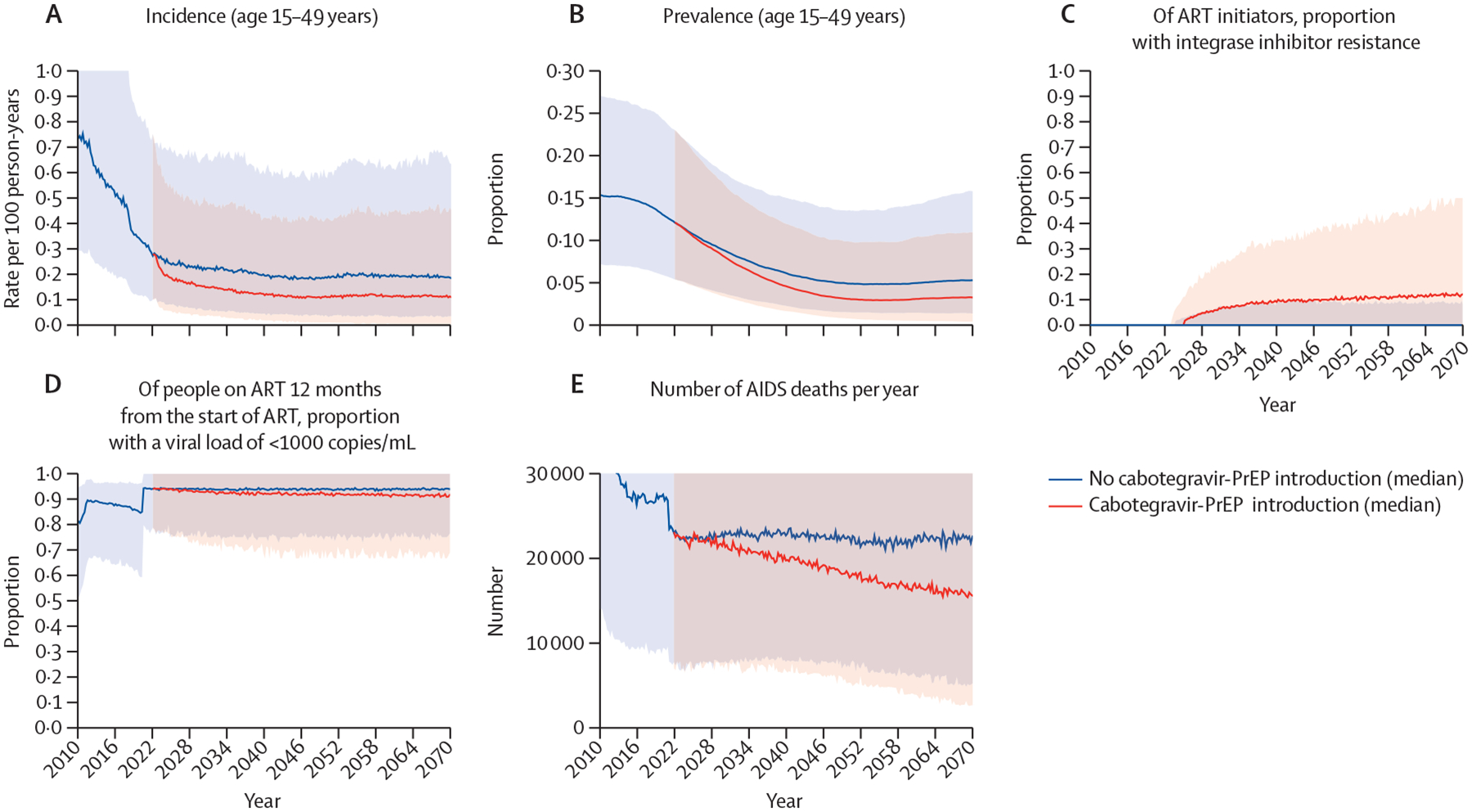
Key outcomes across 50 years according to whether or not cabotegravir-PrEP was introduced (across all 1000 setting-scenarios) ART=antiretroviral therapy. PrEP= pre-exposure prophylaxis.

In the population initiating ART, the proportion with integrase-inhibitor resistance is projected in 20 years to be 1·7% (90% range across setting scenarios 0·0–6·4%; n=200) in the context of a population of 10 million people without cabotegravir-PrEP introduction but 13·1% (4·1–30·9%; n=1141) if cabotegravir-PrEP is introduced with antibody testing (table 3; figure 1). As a consequence, of all people who have been on ART for 12 months, the proportion with viral loads less than 1000 copies per mL is projected to be lower by 2·2% with cabotegravir-PrEP introduction than without (lower by 6·5% to higher by 2·0%) at 20 years. The most influential parameters governing the effect of cabotegravir-PrEP on integrase-inhibitor resistance in ART initiators and on AIDS deaths are shown in the appendix (pp 4–6). As expected, several parameters relating to the incidence and transmission of drug resistance affect the amount of integrase-inhibitor resistance.

If antibody testing is used, the proportion of all people with HIV who have integrase-inhibitor resistance is predicted to be 8·1% (90% range across setting scenarios 2·7–17·6%) in 20 years if cabotegravir-PrEP is introduced, 4·4% (1·0–11·4%) higher than if it is not. Cabotegravir-PrEP introduction is predicted to lower the proportion with viral loads less than 1000 copies per mL among all people with HIV on ART by 0·9% (lower by 2·5% to higher by 0·3%) at 20 years (table 3). Use of NAT tends to mitigate these effects on integrase-inhibitor resistance to a small extent, but there is not a discernible effect of NAT on the proportion of all people on ART with viral loads less than 1000 copies per mL (table 3).

Cabotegravir-PrEP introduction with antibody testing is predicted to lead to 4540 (90% range across setting scenarios 300–13 000) fewer AIDS deaths per year across 50 years (table 4). AIDS deaths are averted with cabotegravir-PrEP introduction in 97% of setting-scenarios. In additional setting-scenarios in which cabotegravir-PrEP was assumed not to lead to integrase-inhibitor resistance, we projected a mean annual reduction in AIDS deaths of 5620 per year with its introduction. Thus, the reduction in our main analysis is approximately 81% of this potential reduction. Consistent with the trend for AIDS deaths, we predict 33 675 DALYs averted per year across 50 years (with 3% discounting) in the main analysis. The effect on DALYs is not discernibly different according to whether NAT is used (table 4) because of the small effect that the HIV testing approach had on the proportion of people on ART with viral loads less than 1000 copies per mL. For AIDS deaths, the benefit is greater (appendix pp 4–6); the more appealing cabotegravir-PrEP is as a PrEP option, the lower the rate of discontinuation of cabotegravir-PrEP, the lower the amount of population adherence (to ART and oral PrEP), and the higher the cabotegravir-PrEP efficacy. We do not find a strong influence of parameters relating to viral load measurement in people on ART or on the rate of switch in ART regimen after virological failure is detected.

Under our base-case assumptions for cabotegravir-PrEP cost, overall HIV programme costs are predicted to be similar with and without cabotegravir-PrEP introduction if antibody testing only is used (table 4). Mean discounted costs across 50 years across setting-scenarios are $151·3 million (ie, $127·4 million for HIV treatment and care, $10·0 million for PrEP drugs and visits, $11·9 million for HIV testing, and $2·0 million for male circumcision) with no cabotegravir-PrEP introduction and $150·7 million (ie, $113·0 million for HIV treatment and care, $20·4 million for PrEP drugs and visits, $15·3 million for HIV testing, and $2·0 million for male circumcision) with cabotegravir-PrEP introduction, assuming the same drug cost for oral PrEP and cabotegravir-PrEP. The treatment and care cost is reduced with cabotegravir-PrEP despite the small increase in use of (more expensive) protease-inhibitor regimens. If NAT is used for people on cabotegravir-PrEP then total mean discounted annual costs are $13·3 million (90% range across setting scenarios –10·9 to 48·1) higher with cabotegravir-PrEP introduction.

The introduction of cabotegravir-PrEP with antibody testing is cost-effective in 82% of setting-scenarios (table 4), whereas in the context of NAT, it is cost-effective in 56–72% of setting-scenarios. With antibody testing, if the cost of cabotegravir is $30 per year instead of $60, cabotegravir-PrEP introduction is cost-effective in 87% of setting-scenarios. Cabotegravir-PrEP is cost-effective in only 52% of setting-scenarios if the cost of the drug and of cabotegravir-PrEP clinic visits are double, at $120 per year each. Cabotegravir-PrEP was cost-effective in an increasing percentage of setting-scenarios with higher HIV incidence in 2022, and in a lower percentage of setting scenarios in which adherence to oral PrEP was higher (table 4).

In a separate sensitivity analysis in which we assume that people will additionally use PrEP if they had condomless sex with at least one short-term partner in the previous 3-month period, but not necessarily in the current 3 month period, cabotegravir-PrEP introduction was cost-effective in 78% of setting-scenarios with our base-case assumptions for cabotegravir-PrEP cost. In another separate sensitivity analyses, the proportion of setting-scenarios in which cabotegravir-PrEP introduction was cost-effective was 73% when the increase in overall PrEP coverage in people with an indication for PrEP was 5–10% (compared with >10% in our main analysis). Finally, we considered an artificial extreme situation in which cabotegravir-PrEP was never used for two consecutive 3-month periods and was never restarted while in the tail period from any previous dose. In this exteme situation, cabotegravir-PrEP introduction did not lead to an increase in overall PrEP use, HIV incidence was not reduced, and AIDS deaths were not averted, but there was an increase in integrase-inhibitor resistance.

## Discussion

We used an established HIV epidemic model to investigate the effects of cabotegravir-PrEP introduction in sub-Saharan Africa. We modelled high uptake of cabotegravir-PrEP, which lead to a substantial decline in HIV incidence and prevalence, but also to increased incidence of integrase-inhibitor resistant virus, resulting in lower viral suppression rates in ART initiators in 20 years, compared with no cabotegravir-PrEP introduction. However, the projected reduction in overall viral suppression among the whole population of people on ART was small and there was a net benefit of cabotegravir-PrEP introduction on numbers of AIDS deaths. Our analysis estimated around a 20% loss of the benefit of cabotegravir-PrEP on reducing AIDS deaths due to integrase-inhibitor resistance. Cabotegravir-PrEP introduction is likely to be cost-effective if it can be delivered at a similar fully loaded cost (including drug, supply chain, clinic visits, and HIV rapid antibody testing) as oral PrEP. The potential manufacturing cost of cabotegravir-PrEP by generic suppliers (excluding CapEx and development costs, which are accounted for separately) has been estimated at approximately $16–23 per patient per year, which is lower than the annual price of oral PrEP commodities based on reference pricing from the Global Fund to Fight AIDS, Tuberculosis and Malaria^32^ and USAID (ie, the USAID Global Health Supply Chain Program product e-Catalog). This price is also lower than our base-case assumption (ie, $60 per year including supply chain) suggesting a high probability that cabotegravir-PrEP could be a beneficial and cost-effective prevention option through generic production.^29^ Our findings on cost-effectiveness are broadly in line with those from a recent evaluation in the context of South Africa.^33^

The small number of HIV infections in the HPTN 083 and HPTN 084 studies mean substantial uncertainty remains about risk of emergence of resistance mutations in the context of cabotegravir-PrEP use and to what degree these mutations will affect HIV treatment efficacy.^8–11^ Nevertheless, incorporating the uncertainty, the probability of there being a net beneficial effect on AIDS deaths across our setting-scenarios was 97%, suggesting that the benefits of cabotegravir-PrEP introduction are likely to outweigh the risks and harms.

We considered potential benefits of NAT for HIV to reduce the chance that a person with early HIV infection starts on cabotegravir-PrEP and to potentially allow early detection of breakthrough infections. We found that integrase-inhibitor resistance prevalence would be reduced among those starting ART, and viral suppression prevalence at 12 months from ART initiation would be increased, but that effects on viral suppression in the whole population of people on ART were small and we were not able to discern a benefit of NAT on AIDS deaths. Given its additional unit cost, we could not find any evidence to suggest that NAT would be cost-effective. Additionally, requiring NAT could make the scale-up of cabotegravir-PrEP unfeasible and likely subject to additional operational costs in most sub-Saharan African countries. Challenges with NAT include limited availability of products as only one product (ie, Aptima) is regulated and approved for adult diagnosis, limited number of sites and personnel that would be needed to implement and achieve the sensitivity modelled, long turnaround times for test results, and limited ability to procure sufficient tests and reagents to meet the scale-up demands modelled. Use of laboratory-based antibody-antigen-based testing also has feasibility concerns with only small gains in sensitivity. Likewise, antibody–antigen rapid tests could provide small gains in sensitivity but have not shown evidence that they can improve the time of detection and diagnose acute HIV infection. Antibody-only rapid testing appears to be sufficient.

The introduction of oral PrEP raised concern about the emergence of drug resistance to tenofovir and lamivudine.^34,35^ Modelling at the time suggested that oral PrEP would lead to an increase in the proportion of people with HIV who carried drug resistance mutations but, contrary to our finding for cabotegravir-PrEP, a decrease in the absolute number with drug resistance.^35^ Concerns about drug resistance due to oral PrEP have been allayed by evidence of high efficacy of ART with dolutegravir, tenofovir, and lamivudine even if resistance to tenofovir or lamivudine is present.^36^ The reliance on the high efficacy of dolutegravir in such regimens reinforces the seriousness of the possibility of integrase-inhibitor resistance emergence. A further source of uncertainty that emphasises the need for caution is the scarce data on integrase-inhibitor mutations in the context of viral subtypes, which are most common in sub-Saharan Africa. Moreover, other second-line integrase-inhibitors, such as bictegravir, could well be similarly compromised. WHO recommends monitoring amounts of integrase-inhibitor drug resistance, and cabotegravir-PrEP introduction enhances this need.^37,38^ Monitoring of viral load response to dolutegravir-containing regimens is also important, and an increase in the risk of virological failure could erode confidence in treatment programmes. Our results suggest that fears of resistance should not delay the introduction of cabotegravir-PrEP, but that the development and roll-out of a long-acting PrEP that is not susceptible to integrase-inhibitor drug resistance is a priority.

We modelled scale-up of cabotegravir-PrEP with availability for all adults. Although our results suggest scale-up is likely to prove a cost-effective approach, the short-term budget effect also needs consideration and countries will need to judge the rate of scale-up and the timing of extending availability beyond adolescent girls and young women, sex workers, and other groups at high risk, such as people who inject drugs and men who have sex with men.

Our study has limitations. Our model simulated a population in 3-monthly time steps, which does not coincide with the current recommendation of cabotegravir-PrEP injections every 2 months. Although we do not expect this difference to have a substantial effect on our inferences, alternative model structures with shorter time steps could be used to replicate our findings. Moreover, 3-monthly dosing might be evaluated in the future for women. Although most transmission in sub-Saharan Africa occurs through heterosexual intercourse, with HIV prevalence generally higher in women than men (table 1), a limitation is that we did not model sex between men as a part of this analysis. Further, we did not explicitly model some other key populations (eg, people who use injection drugs), although we did consider that some people are less likely to have access to testing and PrEP for structural reasons. We assume that cabotegravir-PrEP use will be risk-informed and that there are high levels of adherence to oral PrEP when indicated. It remains uncertain if such risk-informed use will be possible for most people, although open label studies encouragingly suggest that oral PrEP use is concentrated in periods of high risk leading to disproportionate reductions in incidence.^39,40^ Finally, we considered that presence of other sexually transmitted infections increases HIV acquisition risk, but we did not model the transmission of sexually transmitted infections dynamically, which could conceivably affect our results.

In summary, cabotegravir-PrEP introduction is likely to result in net reductions in AIDS deaths and DALYs in addition to reductions in HIV incidence, but there is predicted to be an increased incidence of integrase-inhibitor resistance. Cabotegravir-PrEP is predicted to be cost-effective if delivered at the same cost as oral-PrEP or lower.

## Supplementary Material

Appendix

## Figures and Tables

**Table 1:** Description of setting scenarios in 2022, based on n=1000 setting-scenarios

	Model median (90% range)	Examples of observed data*
HIV prevalence in women, men aged 15–49 years	Women 14·8% (6·3–28·5%), men 9·1% (4·0–17·4%)	Zimbabwe 2016 16%, 11%; 2020 15%, 9% Tanzania 2017 6%, 3% Uganda 2017 8%, 4% Lesotho 2017 30%, 19% Eswatini 2017 34%, 19% Malawi 2016 12%, 8%; 2020 10%, 6% Namibia 2017 15%, 8% Zambia 2016 14%, 8% Cameroon 2017 5%, 2%; 2018 3%, 2% Côte d’Ivoire 2017–18 4%, 1%
HIV incidence in women, men (per 100 person years; aged 15–49 years)	0·57% (0·23–1·45%)	Malawi 2016 0·44, 0·22; 2020 0·31, 0·15 Zambia 2016 1·00, 0·28 Zimbabwe 2016 0·57, 0·30; 2020 0·67, 0·23 Lesotho 2017 1·31, 1·05 Namibia 2016 0·66,0·15 Eswatini 2017 1·73, 0·85 Tanzania 2017 0·34, 0·14 Cameroon 2017 0·40, 0·08
Proportion of women, men diagnosed with HIV	89% (80–95%)	Malawi 2016 80%; 72%; 2020 90%, 85% Zambia 2016 73%, 69% Zimbabwe 2016 80%, 72%; 2020; 88%, 84% Namibia 2017 83%, 71% Tanzania 2017 65%, 52% Ethiopia 2018 83%, 70% Côte d’Ivoire 2017–18 43%, 24% Cameroon 2017 58%, 51%
Proportion of women, men diagnosed with HIV on ART	90% (71–96%)	Lesotho 2016–17 92%, 92% †South Africa 2017 71% Eswatini 2016–17 88%, 90% Namibia 2017 96%, 94% Zambia 2016 87%, 88% Tanzania 2016–17 95%, 90% Ethiopia 96%, 99% Malawi 2016 93%, 89%; 2020 98%, 97% Uganda 2016–17 90%, 85% Cameroon 2017 93%, 94% Zimbabwe 2016 89%, 88%; 2020 98%, 96% Côte d’Ivoire 2017–18 93%, 71%
Proportion of all people with HIV with VL <1000 copies/mL	72% (51–84%)	Zambia 2016 59% Malawi 2016 68%; 2020 87% Zimbabwe 2016 60%; 2020 76% Eswatini 2017 73% Lesotho 2017 68% Tanzania 2017 52% Uganda 2017 60% Namibia 2017 77% Ethiopia 2018 70% Côte d’Ivoire 2017–18 40% Cameroon 2017 47%
Prevalence of VL ≥1000 copies/mL among all adults	3·9% (1·7–8·3%)	Zambia 2016 4·8% (aged 15–59 years) Namibia 2017 2·8% (aged 15–64 years) Malawi 2015–16 3·4% (aged 15–64 years); 2020 1·2% Zimbabwe 2016 5·7% (aged 15–64 years); 2020 3·1% (aged >15 years) Côte d’Ivoire 2018 1·7% (aged 15–64 years) Eswatini 2017 7·3% (aged >15 years) Lesotho 2018 8·3% (aged 15–59 years)
Proportion of men, women on ART with VL <1000 copies/mL	93% (79–99%)	Zambia 2016 90%, 88% Malawi 2016 92%, 90%; 2020 97%, 97% Zimbabwe 2016 88%, 84%; 2020 91%, 89% Namibia 2017 90%, 92% Tanzania 2017 83%, 89% Ethiopia 2018 87%, 95% Côte d’Ivoire 2017–18 78%, 65% Cameroon 2017 80%, 81%

ART=antiretroviral therapy. VL=HIV RNA viral load.

* All observed data from are from Population-Based HIV Impact Assessment surveys unless stated. Of model runs, those with HIV incidence at 15–49 year olds was less than 0·15 per 100 person years or HIV prevalence in 15–49 year olds was more than 25% in mid-2022, or for which the increase in the proportion of people with an indication for pre-exposure prophylaxis who were on it (mean over 20 years) with long-acting cabotegravir pre-exposure prophylaxis introduction was less than 10%, were excluded (437 runs were excluded due to fulfilling one of these criteria, 381 of which were excluded due to the third condition).

† The South Africa estimate is from the Fifth South African National HIV Prevalence, Incidence, Behaviour, and Communication Survey.^17^

**Table 2:** 20-year outcomes for long-acting cabotegravir-PrEP use assumptions and effects on HIV of long-acting cabotegravir-PrEP introduction

	No cabotegravir-PrEP	Introduction of cabotegravir-PrEP	Difference (or % reduction)
**Outputs illustrating assumptions on cabotegravir-PrEP introduction and use**			
Proportion of people with indication for PrEP who are on PrEP*	28% (12–44%)	46% (25–65%)	18% (10–27%)
Proportion of people aged 15–64 years on PrEP*	1·5% (0·4–3·6%)	2·6% (0·8–6·1%)	1·0% (0·2–2·7%)
Number of people in a population of 10 million adults taking PrEP in any given 3 month period*	155 000 (43 000–365 000)	265 000 (78 000–632 000)	109 000 (26 000–288 000)
Proportion of people on PrEP using cabotegravir-PrEP*	0%	71% (47–86%)	··
Proportion of people aged older than 15 years who had ever taken PrEP†	17% (7–28%)	25% (10–41%)	8% (3–13%)
Proportion of people using PrEP who have taken PrEP for ≥5 years†	3% (0–13%)	3% (0–12%)	0% (–4 to 5%)
**Effect of cabotegravir use on HIV incidence and prevalence**			
HIV incidence in people aged 15–49 years (per 100 person years)*	0·54 (0·16–1·26)	0·38 (0·11–0·91)	29% reduction (6–52%)
HIV incidence (per 100 person years) in people on PrEP*	4·0 (0·7–11·3)	1·6 (0·3–4·4)	−2·4 (−7·3 to −0·3)
Counter-factual HIV incidence in people who take PrEP as it would have been without PrEP*‡	13·6 (3·2–31·2)	13·8 (3·1–30·8)	
Number of births of children born with HIV per year*§	7200 (1400–20 200)	5700 (1100–15 800)	−1470 (200 to −4700)
HIV prevalence in people aged 15–49 years†	6·6% (1·9–14·7%)	4·9% (1·4–11·4%)	26% reduction (4–47%)
Number of people living with HIV†	1 462 000 (560 000–2 855 000)	1 229 000 (466 000–2 369 000)	16% reduction (3–29%)

Data are mean across setting scenarios (90% range across setting scenarios). PrEP=pre-exposure prophylaxis.

* Means calculated over 20 years (across all the 3 month periods in the 20 years) with 90% ranges across the setting scenarios.

† Means calculated at 20 years (across all the 3 month periods in the 7 years centered around 20 years, 16·5 years to 23·5 years), with 90% ranges across the setting scenarios.

‡ If PrEP efficacy=0.

§ Absolute numbers relate to a population containing 10 million adults.

**Table 3:** Effects of long-acting cabotegravir-PrEP introduction on integrase-inhibitor drug resistance across 20 years with different HIV testing approaches

	No cabotegravir-PrEP	Introduction of cabotegravir-PrEP	Difference
**Proportion of people on cabotegravir-PrEP who have HIV** *			
Antibody testing only	··	0·67% (0·07–1·87%)	0·67% (0·07–1·87%)
NAT at cabotegravir-PrEP initiation and re-initiation only†	··	0·22% (0·03–0·63%)	0·22% (0·03–0·63%)
NAT throughout cabotegravir-PrEP†	··	0·16% (0·02–0·51%)	0·16% (0·02–0·51%)
**Proportion of ART initiators with integrase-inhibitor resistance** ‡			
Antibody testing only	1·7% (0·0–6·4%)	13·1% (4·1–30·9%)	11·4% (3·0–26·4%)
NAT at cabotegravir-PrEP initiation and re-initiation only†	2·0% (0·0–7·3%)	12·5% (3·8–20·3%)	10·5% (3·1–21·9%)
NAT throughout cabotegravir-PrEP†	1·5% (0·0–5·8%)	8·3% (2·2–21·4%)	6·6% (1·7–18·4%)
**Number of ART initiators per 3 months with integrase-inhibitor resistance** ‡ §			
Antibody testing only	200 (0–850)	1141 (125–3612)	929 (81–3118)
NAT at cabotegravir-PrEP initiation and re-initiation only†	240 (0–990)	1006 (136–2809)	768 (86–2274)
NAT throughout cabotegravir-PrEP†	204 (0–852)	703 (68–2649)	499 (22–1974)
**Proportion of all people with HIV with integrase-inhibitor resistance** ‡			
Antibody testing only	3·7% (0·8–10·5%)	8·1% (2·7–17·6%)	4·4% (1·0–11·4%)
NAT at cabotegravir-PrEP initiation and re-initiation only†	4·4% (0·7–12·4%)	8·4% (2·6–18·7%)	4·0% (0·9–8·5%)
NAT throughout cabotegravir-PrEP†	4·0% (0·7–10·6%)	6·9% (2·0–14·2%)	2·9% (0·6–7·4%)
**Total number of people with integrase-inhibitor resistant HIV** ‡ §			
Antibody testing only	56 200 (7700–177 400)	106 000 (20 500–276 800)	49 600 (2400–162 000)
NAT at cabotegravir-PrEP initiation and re-initiation only†	65 000 (6300–214 000)	104 400 (20 000–290 500)	39 700 (0–119 200)
NAT testing throughout cabotegravir-PrEP†	58 300 (7000–191 000)	85 100 (13 800–248 800)	26 800 (−3500 to 93 300)
**Number of people infected with integrase-inhibitor resistant virus** ‡ §			
Antibody testing only	11 400 (400–40 700)	27 100 (1350–106 300)	15 700 (−3500 to 70 100)
NAT at cabotegravir-PrEP initiation and re-initiation only†	12 800 (400–51 900)	23 800 (1700–84 900)	11 000 (−5200 to 47 950)
NAT throughout cabotegravir-PrEP†	11 000 (200–40 400)	17 400 (600–70 900)	6500 (−7000 to 37 100)
**Of people on ART at 12 months after starting ART, proportion with VL <1000 copies per mL** ‡			
Antibody testing only	92% (80–97%)	90% (77–96%)	−2·2% (−6·5 to 2·0%)
NAT testing at Cab-PrEP initiation and re-initiation only†	92% (79–98%)	90% (76–97%)	−2·1% (−6·4 to 1·5%)
NAT testing throughout Cab-PrEP†	92% (80–97%)	91% (78–97%)	−1·4% (−5·6–1·9%)
**Proportion of all people on ART with VL <1000 copies per mL** ‡			
Antibody testing only	94% (87–98%)	94% (86–98%)	−0·9% (−2·5 to 0·3%)
NAT testing at Cab-PrEP initiation and re-initiation only†	94% (84–98%)	93% (84–98%)	−0·7% (−2·2 to 0·6%)
NAT testing throughout Cab-PrEP†	94% (88–98%)	94% (88–98%)	−0·5% (−1·8 to 0·5%)
**Proportion of people on ART on boosted protease inhibitor regimen** ‡			
Antibody testing only	4% (0–10%)	4% (0–11%)	1% (0–2%)
NAT at cabotegravir-PrEP initiation and re-initiation only†	4% (0–11%)	5% (0–12%)	1% (0–2%)
NAT throughout cabotegravir-PrEP†	4% (0–10%)	4% (0–12%)	0% (0–2%)

Data are mean across setting scenarios (90% range). PrEP=pre-exposure prophylaxis. NAT=nucleic acid-based HIV diagnostic testing. ART=antiretroviral therapy. VL=HIV RNA viral load.

* Means calculated over 20 years (across all the 3 month periods in the 20 years), with 90% ranges across the setting scenarios.

† NAT testing not used for people on oral PrEP.

‡ Means calculated at 20 years (across all the 3 month periods in the 7 years centered around 20 years, 16·5 years to 23·5 years), with 90% ranges across the setting scenarios.

§ Absolute numbers relate to a population containing 10 million adults.

**Table 4:** Effects of long-acting cabotegravir-PrEP introduction on AIDS deaths, DALY, costs, and net DALYs across 50 years with different HIV testing approaches

	No cabotegravir-PrEP	Introduction of cabotegravir-PrEP	Difference (if applicable)
**Mean number of AIDS deaths per year**			
Antibody testing only	28 460 (9520–64 170)	23 920 (8100–53 800)	−4540 (−13 000 to −300)
NAT at cabotegravir-PrEP initiation and re-initiation only*	26 580 (9180–57 600)	21 840 (7630–47 300)	−4750 (−13 500 to −450)
NAT throughout cabotegravir-PrEP*	26 340 (9250–57 700)	21 920 (7800–46 700)	−4410 (−12 460 to −510)
**Percentage of setting-scenarios in which AIDS deaths were averted**			
Antibody testing only	··	97%	··
NAT at cabotegravir-PrEP initiation and re-initiation only	··	97%	··
NAT throughout cabotegravir-PrEP	··	99%	··
**DALYs averted per year (mean per year, discounted at 3% per year)**			
Antibody testing only	··	33 675	··
NAT at cabotegravir-PrEP initiation and re-initiation only	··	36 540	··
NAT throughout cabotegravir-PrEP	··	32 640	··
**HIV programme costs per year (mean US$ million per year, discounted at 3% per year; $60 drug cost, including supply chain)** †			
Antibody testing only	151·3 (73·5–263·1)	150·7 (74·8–257·5)	−0·6 (−18·1 to 18·7)
NAT at cabotegravir-PrEP initiation and re-initiation only	148·3 (65·3–251·8)	155·2 (71·7–270·7)	6·8 (−12·5 to 31·3)
NAT testing throughout cabotegravir-PrEP	143·6 (60·6–260·1)	156·9 (66·8–290·8)	13·3 (−10·9 to 48·1)
**Net DALYs averted (US$60 drug cost)**			
Antibody testing only	··	34 780	··
NAT at cabotegravir-PrEP initiation and re-initiation only	··	22 920	··
NAT throughout cabotegravir-PrEP	··	5970	··
**Percentage of setting-scenarios in which Cab-PrEP introduction is cost-effective (US$60 drug cost)**			
Antibody testing only	··	82%	··
NAT at cabotegravir-PrEP initiation and re-initiation only	··	72%	··
NAT throughout cabotegravir-PrEP	··	56%	··
**Percentage of setting-scenarios in which cabotegravir-PrEP introduction is cost-effective (antibody testing only) according to overall incidence in people aged 15–49 years**			
<0·5 per 100 person-years	··	73%	··
0·5–<1·0 per 100 person-years	··	87%	··
≥1·0 per 100 person-years	··	92%	··
**Percentage of setting-scenarios in which cabotegravir-PrEP introduction is cost-effective (antibody testing only) according to oral PrEP adherence level**‡ **(% at least 80% adherent)**			
<70%	··	86%	··
70–79%	··	83%	··
80–89%	··	83%	··
≥90%	··	67%	··
**Percentage of setting-scenarios in which cabotegravir-PrEP introduction is cost-effective with alternative annual cost of cabotegravir-PrEP, including delivery (antibody testing only; base case US$144)**			
$114	··	87%	··
$264	··	52%	··

Data are mean across setting-scenarios (90% range) or % of setting scenarios. PrEP=pre-exposure prophylaxis. NAT=nucleic acid-based HIV diagnostic testing. DALY=disability-adjusted life years.

* NAT not used for people on oral PrEP.

† Other costs beyond PrEP and treatment and care include voluntary medical male circumcision and HIV testing.

‡ Mean across 20 years.

## Research in context

### Evidence before this study

After showing high efficacy in randomised trials, long-acting injectable cabotegravir pre-exposure prophylaxis (PrEP) is being considered for introduction in sub-Saharan Africa as an additional PrEP option for HIV prevention. There is concern, however, that cabotegravir-PrEP use could lead to increases in the prevalence of integrase-inhibitor resistant virus, which could undermine current treatment programmes that rely on a closely related drug, dolutegravir. We searched Web of Science on June 25, 2022, with the terms “cabotegravir” and “resistance” and “pre-exposure prophylaxis” and identified no modelling studies for sub-Saharan Africa that have attempted to project the net effects of cabotegravir-PrEP introduction on AIDS deaths and disability adjusted life years, including the effects of drug resistance.

### Added value of this study

We jointly modelled the beneficial effects of cabotegravir-PrEP on HIV incidence and the negative effect on integrase-inhibitor drug resistance. Projecting the combined effects of cabotegravir-PrEP we found a net benefit of its introduction on numbers of AIDS deaths. We further found that cabotegravir-PrEP introduction is likely to be cost-effective if the drug can be delivered at a similar fully-loaded cost as the oral PrEP drug (ie, USD$60 per year including supply chain).

### Implications of all the available evidence

Cabotegravir-PrEP introduction has the potential to have substantial net benefits on AIDS deaths in addition to reductions in incidence if adequately used in a risk-informed way. It is predicted to be cost-effective if delivered at the same cost as oral PrEP.
